# Supplementary material for: Multi‐dataset identification of innovative feature genes and molecular mechanisms in keratoconus
Source: J Cell Mol Med. 2024 Sep 19;28(18):e70079. doi: 10.1111/jcmm.70079 (PMC11412914; doi:10.1111/jcmm.70079)
Supplement: Supplementary file 2 — Material S2. [file JCMM-28-e70079-s002.zip › 7.GSEA analysis/3.DBNDD1/KEGG_CYTOKINE_CYTOKINE_RECEPTOR_INTERACTION.html]

Details for gene set KEGG\_CYTOKINE\_CYTOKINE\_RECEPTOR\_INTERACTION[GSEA]

|  || Dataset | DBNDD1.group.cls#h\_versus\_l |
| Phenotype | group.cls#h\_versus\_l |
| Upregulated in class | l |
| GeneSet | KEGG\_CYTOKINE\_CYTOKINE\_RECEPTOR\_INTERACTION |
| Enrichment Score (ES) | -0.4307543 |
| Normalized Enrichment Score (NES) | -1.311059 |
| Nominal p-value | 0.098425195 |
| FDR q-value | 0.78409004 |
| FWER p-Value | 0.958 |
Table: GSEA Results Summary

  

Fig 1: Enrichment plot: KEGG\_CYTOKINE\_CYTOKINE\_RECEPTOR\_INTERACTION      
 Profile of the Running ES Score & Positions of GeneSet Members on the Rank Ordered List

  

| SYMBOL | TITLE | RANK IN GENE LIST | RANK METRIC SCORE | RUNNING ES | CORE ENRICHMENT || 1 | GHR | na | 396 | 0.477 | -0.0000 | No |
| 2 | IFNA14 | na | 550 | 0.438 | 0.0061 | No |
| 3 | IL21 | na | 937 | 0.390 | 0.0042 | No |
| 4 | IFNL3 | na | 1217 | 0.360 | 0.0048 | No |
| 5 | IL3RA | na | 1219 | 0.360 | 0.0134 | No |
| 6 | IL12A | na | 1227 | 0.359 | 0.0219 | No |
| 7 | IFNA6 | na | 1774 | 0.315 | 0.0136 | No |
| 8 | IL9 | na | 1908 | 0.307 | 0.0171 | No |
| 9 | IL26 | na | 2135 | 0.291 | 0.0175 | No |
| 10 | IL4R | na | 2229 | 0.286 | 0.0217 | No |
| 11 | NGFR | na | 2398 | 0.277 | 0.0235 | No |
| 12 | IL7R | na | 2510 | 0.272 | 0.0268 | No |
| 13 | GH1 | na | 2516 | 0.272 | 0.0332 | No |
| 14 | PDGFRA | na | 3213 | 0.243 | 0.0188 | No |
| 15 | IL2RB | na | 3295 | 0.239 | 0.0222 | No |
| 16 | CXCR2 | na | 3338 | 0.238 | 0.0267 | No |
| 17 | IL17B | na | 3434 | 0.235 | 0.0296 | No |
| 18 | HGF | na | 3455 | 0.233 | 0.0346 | No |
| 19 | IL17RB | na | 3531 | 0.230 | 0.0380 | No |
| 20 | AMHR2 | na | 3557 | 0.229 | 0.0428 | No |
| 21 | IL3 | na | 3952 | 0.215 | 0.0365 | No |
| 22 | CCR10 | na | 4137 | 0.209 | 0.0362 | No |
| 23 | BMPR1B | na | 4162 | 0.208 | 0.0405 | No |
| 24 | IFNA16 | na | 4203 | 0.207 | 0.0443 | No |
| 25 | CCL2 | na | 4419 | 0.199 | 0.0428 | No |
| 26 | TGFBR2 | na | 4515 | 0.197 | 0.0448 | No |
| 27 | FLT3 | na | 4749 | 0.188 | 0.0426 | No |
| 28 | PF4 | na | 4760 | 0.188 | 0.0468 | No |
| 29 | TNFSF14 | na | 5346 | 0.169 | 0.0339 | No |
| 30 | TNFRSF19 | na | 5363 | 0.168 | 0.0374 | No |
| 31 | EPO | na | 5420 | 0.167 | 0.0398 | No |
| 32 | IL5RA | na | 6076 | 0.148 | 0.0244 | No |
| 33 | LEP | na | 6081 | 0.148 | 0.0278 | No |
| 34 | CCR2 | na | 6130 | 0.147 | 0.0300 | No |
| 35 | PPBP | na | 6173 | 0.146 | 0.0323 | No |
| 36 | TNFRSF10C | na | 6465 | 0.138 | 0.0271 | No |
| 37 | IL23R | na | 6596 | 0.134 | 0.0266 | No |
| 38 | TNFRSF13C | na | 7128 | 0.122 | 0.0141 | No |
| 39 | TPO | na | 7176 | 0.120 | 0.0156 | No |
| 40 | ACVR2B | na | 7261 | 0.119 | 0.0160 | No |
| 41 | IL7 | na | 7827 | 0.107 | 0.0022 | No |
| 42 | CCL26 | na | 7877 | 0.106 | 0.0033 | No |
| 43 | IFNL2 | na | 7969 | 0.104 | 0.0032 | No |
| 44 | IFNE | na | 8252 | 0.099 | -0.0026 | No |
| 45 | CD70 | na | 8665 | 0.092 | -0.0123 | No |
| 46 | FASLG | na | 8688 | 0.091 | -0.0108 | No |
| 47 | TNFRSF13B | na | 8984 | 0.086 | -0.0173 | No |
| 48 | PDGFA | na | 9340 | 0.081 | -0.0256 | No |
| 49 | CCL15 | na | 9778 | 0.074 | -0.0365 | No |
| 50 | CCL23 | na | 10022 | 0.071 | -0.0419 | No |
| 51 | PDGFRB | na | 10046 | 0.071 | -0.0408 | No |
| 52 | CCL3L3 | na | 10246 | 0.068 | -0.0450 | No |
| 53 | IL2RG | na | 10291 | 0.067 | -0.0446 | No |
| 54 | CCR4 | na | 10530 | 0.064 | -0.0500 | No |
| 55 | TNFRSF25 | na | 10540 | 0.064 | -0.0487 | No |
| 56 | ACVRL1 | na | 10841 | 0.060 | -0.0560 | No |
| 57 | IL15RA | na | 10985 | 0.058 | -0.0587 | No |
| 58 | TNFSF15 | na | 11015 | 0.058 | -0.0582 | No |
| 59 | TNFRSF11B | na | 11048 | 0.058 | -0.0577 | No |
| 60 | TNFSF13B | na | 11445 | 0.053 | -0.0680 | No |
| 61 | PLEKHO2 | na | 11455 | 0.053 | -0.0669 | No |
| 62 | IFNL1 | na | 11477 | 0.052 | -0.0663 | No |
| 63 | IL11RA | na | 11720 | 0.050 | -0.0721 | No |
| 64 | CXCR5 | na | 11759 | 0.050 | -0.0720 | No |
| 65 | BMPR2 | na | 12004 | 0.047 | -0.0780 | No |
| 66 | IL20RA | na | 12556 | 0.042 | -0.0930 | No |
| 67 | XCR1 | na | 12568 | 0.042 | -0.0923 | No |
| 68 | TNFSF13 | na | 12671 | 0.041 | -0.0942 | No |
| 69 | INHBC | na | 12851 | 0.039 | -0.0985 | No |
| 70 | GH2 | na | 12931 | 0.039 | -0.0998 | No |
| 71 | IFNA2 | na | 13108 | 0.037 | -0.1041 | No |
| 72 | TNFSF9 | na | 13237 | 0.036 | -0.1069 | No |
| 73 | CCL17 | na | 13259 | 0.036 | -0.1066 | No |
| 74 | TGFB3 | na | 13624 | 0.033 | -0.1164 | No |
| 75 | IL22 | na | 13637 | 0.033 | -0.1160 | No |
| 76 | CCR6 | na | 13914 | 0.031 | -0.1232 | No |
| 77 | CCL11 | na | 14105 | 0.029 | -0.1280 | No |
| 78 | CTF1 | na | 14147 | 0.029 | -0.1285 | No |
| 79 | LTBR | na | 14375 | 0.027 | -0.1345 | No |
| 80 | FLT4 | na | 14460 | 0.027 | -0.1363 | No |
| 81 | CCR5 | na | 14673 | 0.025 | -0.1418 | No |
| 82 | CRLF2 | na | 14756 | 0.025 | -0.1436 | No |
| 83 | IL18R1 | na | 14876 | 0.024 | -0.1465 | No |
| 84 | CXCL5 | na | 15230 | 0.021 | -0.1562 | No |
| 85 | FLT3LG | na | 15748 | 0.018 | -0.1708 | No |
| 86 | FLT1 | na | 15887 | 0.017 | -0.1744 | No |
| 87 | IFNB1 | na | 15923 | 0.016 | -0.1750 | No |
| 88 | TNFRSF14 | na | 16507 | 0.013 | -0.1916 | No |
| 89 | CCL4L2 | na | 16968 | 0.010 | -0.2047 | No |
| 90 | TSLP | na | 16971 | 0.010 | -0.2046 | No |
| 91 | TNFSF4 | na | 17180 | 0.008 | -0.2104 | No |
| 92 | CNTFR | na | 17407 | 0.007 | -0.2168 | No |
| 93 | VEGFB | na | 17569 | 0.006 | -0.2213 | No |
| 94 | CCL18 | na | 17870 | 0.004 | -0.2299 | No |
| 95 | TNFRSF17 | na | 17975 | 0.003 | -0.2329 | No |
| 96 | PRL | na | 18062 | 0.002 | -0.2353 | No |
| 97 | KDR | na | 18209 | 0.001 | -0.2395 | No |
| 98 | IL18 | na | 18323 | 0.001 | -0.2428 | No |
| 99 | PF4V1 | na | 18854 | -0.003 | -0.2581 | No |
| 100 | IL13RA1 | na | 19160 | -0.006 | -0.2668 | No |
| 101 | TGFB2 | na | 19303 | -0.007 | -0.2707 | No |
| 102 | EGF | na | 19333 | -0.007 | -0.2714 | No |
| 103 | KIT | na | 19563 | -0.009 | -0.2778 | No |
| 104 | IL20RB | na | 19694 | -0.010 | -0.2814 | No |
| 105 | IL25 | na | 20148 | -0.014 | -0.2942 | No |
| 106 | TNFRSF10A | na | 20481 | -0.017 | -0.3034 | No |
| 107 | CCL24 | na | 20660 | -0.019 | -0.3081 | No |
| 108 | BMPR1A | na | 20705 | -0.019 | -0.3089 | No |
| 109 | LTA | na | 21038 | -0.022 | -0.3180 | No |
| 110 | CCL1 | na | 21163 | -0.023 | -0.3210 | No |
| 111 | CCL21 | na | 21387 | -0.026 | -0.3269 | No |
| 112 | IL17RA | na | 21406 | -0.026 | -0.3268 | No |
| 113 | IL10RB | na | 21509 | -0.027 | -0.3291 | No |
| 114 | CD40 | na | 21562 | -0.028 | -0.3299 | No |
| 115 | EGFR | na | 22041 | -0.033 | -0.3430 | No |
| 116 | IFNA4 | na | 22069 | -0.033 | -0.3430 | No |
| 117 | ACVR1B | na | 22088 | -0.033 | -0.3428 | No |
| 118 | CXCL14 | na | 22280 | -0.035 | -0.3475 | No |
| 119 | IFNAR1 | na | 22347 | -0.036 | -0.3485 | No |
| 120 | KITLG | na | 22603 | -0.039 | -0.3550 | No |
| 121 | CXCL16 | na | 22604 | -0.039 | -0.3541 | No |
| 122 | IFNGR1 | na | 22616 | -0.039 | -0.3535 | No |
| 123 | TNFRSF10B | na | 22768 | -0.040 | -0.3569 | No |
| 124 | TNFRSF1A | na | 22847 | -0.041 | -0.3581 | No |
| 125 | OSM | na | 23244 | -0.046 | -0.3685 | No |
| 126 | XCL1 | na | 23361 | -0.048 | -0.3707 | No |
| 127 | BMP7 | na | 23507 | -0.050 | -0.3737 | No |
| 128 | IL19 | na | 23793 | -0.054 | -0.3807 | No |
| 129 | CCL16 | na | 23920 | -0.055 | -0.3831 | No |
| 130 | CCR1 | na | 24035 | -0.057 | -0.3850 | No |
| 131 | TNFSF12 | na | 24046 | -0.057 | -0.3839 | No |
| 132 | CXCL2 | na | 24095 | -0.058 | -0.3839 | No |
| 133 | ACVR2A | na | 24371 | -0.061 | -0.3904 | No |
| 134 | TNFSF8 | na | 24416 | -0.062 | -0.3902 | No |
| 135 | TNFSF10 | na | 24501 | -0.063 | -0.3911 | No |
| 136 | CXCR6 | na | 24515 | -0.063 | -0.3900 | No |
| 137 | CSF3R | na | 24553 | -0.064 | -0.3895 | No |
| 138 | FAS | na | 24662 | -0.066 | -0.3911 | No |
| 139 | IL12RB1 | na | 24684 | -0.066 | -0.3901 | No |
| 140 | IL6 | na | 24766 | -0.067 | -0.3908 | No |
| 141 | TNFSF11 | na | 24768 | -0.067 | -0.3892 | No |
| 142 | IL12B | na | 25009 | -0.071 | -0.3945 | No |
| 143 | EDA2R | na | 25072 | -0.072 | -0.3946 | No |
| 144 | CCR8 | na | 25084 | -0.072 | -0.3932 | No |
| 145 | CCL8 | na | 25104 | -0.072 | -0.3920 | No |
| 146 | IL2RA | na | 25190 | -0.073 | -0.3927 | No |
| 147 | IL6ST | na | 25879 | -0.085 | -0.4106 | No |
| 148 | TNFRSF10D | na | 26206 | -0.092 | -0.4179 | No |
| 149 | CCR3 | na | 26257 | -0.093 | -0.4171 | No |
| 150 | TGFBR1 | na | 26390 | -0.095 | -0.4186 | No |
| 151 | AMH | na | 26603 | -0.100 | -0.4224 | No |
| 152 | CXCL12 | na | 26671 | -0.101 | -0.4219 | No |
| 153 | ACVR1 | na | 26895 | -0.105 | -0.4258 | No |
| 154 | CXCL8 | na | 26911 | -0.106 | -0.4237 | No |
| 155 | CD27 | na | 26929 | -0.106 | -0.4217 | No |
| 156 | CSF2RB | na | 26951 | -0.107 | -0.4197 | No |
| 157 | TNF | na | 27164 | -0.111 | -0.4232 | No |
| 158 | CCL28 | na | 27313 | -0.115 | -0.4247 | No |
| 159 | CSF1 | na | 27462 | -0.118 | -0.4262 | No |
| 160 | TNFRSF12A | na | 27569 | -0.120 | -0.4264 | No |
| 161 | IFNGR2 | na | 27570 | -0.120 | -0.4235 | No |
| 162 | EDAR | na | 27822 | -0.127 | -0.4277 | Yes |
| 163 | CSF1R | na | 27843 | -0.127 | -0.4252 | Yes |
| 164 | IL23A | na | 27929 | -0.129 | -0.4246 | Yes |
| 165 | MPL | na | 28023 | -0.132 | -0.4241 | Yes |
| 166 | IL13 | na | 28093 | -0.133 | -0.4229 | Yes |
| 167 | CXCL13 | na | 28109 | -0.134 | -0.4201 | Yes |
| 168 | IL12RB2 | na | 28204 | -0.135 | -0.4196 | Yes |
| 169 | IFNAR2 | na | 28242 | -0.136 | -0.4174 | Yes |
| 170 | IL6R | na | 28280 | -0.137 | -0.4152 | Yes |
| 171 | CXCR3 | na | 28309 | -0.138 | -0.4127 | Yes |
| 172 | TNFRSF4 | na | 28496 | -0.143 | -0.4146 | Yes |
| 173 | IL1B | na | 28762 | -0.150 | -0.4187 | Yes |
| 174 | IL5 | na | 28795 | -0.151 | -0.4160 | Yes |
| 175 | CCL13 | na | 28966 | -0.156 | -0.4172 | Yes |
| 176 | RELT | na | 29040 | -0.158 | -0.4155 | Yes |
| 177 | CCR9 | na | 29179 | -0.162 | -0.4156 | Yes |
| 178 | LEPR | na | 29224 | -0.163 | -0.4130 | Yes |
| 179 | LIFR | na | 29236 | -0.164 | -0.4094 | Yes |
| 180 | CXCL11 | na | 29277 | -0.165 | -0.4066 | Yes |
| 181 | IL2 | na | 29432 | -0.170 | -0.4069 | Yes |
| 182 | MET | na | 29439 | -0.170 | -0.4030 | Yes |
| 183 | GDF5 | na | 29452 | -0.171 | -0.3993 | Yes |
| 184 | IL9R | na | 29538 | -0.174 | -0.3976 | Yes |
| 185 | CCL19 | na | 29544 | -0.174 | -0.3935 | Yes |
| 186 | CCL14 | na | 29709 | -0.179 | -0.3940 | Yes |
| 187 | CCR7 | na | 29717 | -0.180 | -0.3899 | Yes |
| 188 | TNFRSF1B | na | 29728 | -0.180 | -0.3859 | Yes |
| 189 | VEGFA | na | 30038 | -0.189 | -0.3903 | Yes |
| 190 | CSF3 | na | 30125 | -0.192 | -0.3881 | Yes |
| 191 | CLCF1 | na | 30127 | -0.192 | -0.3836 | Yes |
| 192 | CSF2 | na | 30210 | -0.195 | -0.3813 | Yes |
| 193 | INHBE | na | 30262 | -0.196 | -0.3780 | Yes |
| 194 | CXCL3 | na | 30419 | -0.201 | -0.3777 | Yes |
| 195 | CX3CR1 | na | 30440 | -0.202 | -0.3734 | Yes |
| 196 | IFNA8 | na | 30493 | -0.203 | -0.3701 | Yes |
| 197 | IL1R2 | na | 30516 | -0.204 | -0.3658 | Yes |
| 198 | IL20 | na | 30671 | -0.210 | -0.3652 | Yes |
| 199 | CXCL6 | na | 30806 | -0.214 | -0.3640 | Yes |
| 200 | IFNA7 | na | 30811 | -0.214 | -0.3589 | Yes |
| 201 | EDA | na | 30848 | -0.216 | -0.3548 | Yes |
| 202 | TGFB1 | na | 30914 | -0.218 | -0.3515 | Yes |
| 203 | IL15 | na | 31024 | -0.221 | -0.3493 | Yes |
| 204 | IL1A | na | 31036 | -0.222 | -0.3443 | Yes |
| 205 | IL1RAP | na | 31082 | -0.223 | -0.3402 | Yes |
| 206 | CCL20 | na | 31140 | -0.226 | -0.3365 | Yes |
| 207 | EPOR | na | 31178 | -0.227 | -0.3321 | Yes |
| 208 | IL10 | na | 31214 | -0.229 | -0.3276 | Yes |
| 209 | CCL3 | na | 31224 | -0.229 | -0.3223 | Yes |
| 210 | BMP2 | na | 31395 | -0.236 | -0.3216 | Yes |
| 211 | IL22RA1 | na | 31485 | -0.239 | -0.3184 | Yes |
| 212 | CD40LG | na | 31492 | -0.240 | -0.3128 | Yes |
| 213 | VEGFC | na | 31739 | -0.251 | -0.3140 | Yes |
| 214 | IFNW1 | na | 31807 | -0.255 | -0.3098 | Yes |
| 215 | CCL7 | na | 31858 | -0.256 | -0.3051 | Yes |
| 216 | CCL25 | na | 32045 | -0.266 | -0.3041 | Yes |
| 217 | CCL5 | na | 32225 | -0.277 | -0.3026 | Yes |
| 218 | TNFRSF21 | na | 32329 | -0.284 | -0.2988 | Yes |
| 219 | LIF | na | 32371 | -0.286 | -0.2931 | Yes |
| 220 | TNFRSF11A | na | 32637 | -0.303 | -0.2935 | Yes |
| 221 | INHBB | na | 32766 | -0.312 | -0.2897 | Yes |
| 222 | CX3CL1 | na | 32800 | -0.314 | -0.2831 | Yes |
| 223 | IL21R | na | 32838 | -0.316 | -0.2766 | Yes |
| 224 | IFNA10 | na | 32985 | -0.328 | -0.2730 | Yes |
| 225 | TNFRSF8 | na | 32987 | -0.328 | -0.2651 | Yes |
| 226 | TNFRSF9 | na | 32988 | -0.328 | -0.2573 | Yes |
| 227 | CCL22 | na | 32998 | -0.328 | -0.2496 | Yes |
| 228 | CXCR4 | na | 33022 | -0.330 | -0.2424 | Yes |
| 229 | INHBA | na | 33048 | -0.332 | -0.2351 | Yes |
| 230 | TNFRSF6B | na | 33075 | -0.334 | -0.2278 | Yes |
| 231 | CNTF | na | 33174 | -0.342 | -0.2225 | Yes |
| 232 | IL1R1 | na | 33185 | -0.343 | -0.2145 | Yes |
| 233 | CXCL1 | na | 33274 | -0.350 | -0.2086 | Yes |
| 234 | IL18RAP | na | 33339 | -0.356 | -0.2019 | Yes |
| 235 | CXCR1 | na | 33350 | -0.357 | -0.1936 | Yes |
| 236 | TNFSF18 | na | 33364 | -0.358 | -0.1854 | Yes |
| 237 | TNFRSF18 | na | 33379 | -0.359 | -0.1772 | Yes |
| 238 | CSF2RA | na | 33430 | -0.364 | -0.1699 | Yes |
| 239 | PRLR | na | 33449 | -0.366 | -0.1616 | Yes |
| 240 | IFNLR1 | na | 33526 | -0.372 | -0.1549 | Yes |
| 241 | IL24 | na | 33613 | -0.381 | -0.1483 | Yes |
| 242 | IL4 | na | 33666 | -0.386 | -0.1405 | Yes |
| 243 | CCL27 | na | 33678 | -0.388 | -0.1315 | Yes |
| 244 | IFNG | na | 33717 | -0.393 | -0.1231 | Yes |
| 245 | PDGFB | na | 34011 | -0.431 | -0.1213 | Yes |
| 246 | IFNA21 | na | 34135 | -0.449 | -0.1141 | Yes |
| 247 | IFNK | na | 34142 | -0.450 | -0.1034 | Yes |
| 248 | PDGFC | na | 34154 | -0.452 | -0.0929 | Yes |
| 249 | CXCL9 | na | 34407 | -0.517 | -0.0878 | Yes |
| 250 | IL17A | na | 34413 | -0.519 | -0.0754 | Yes |
| 251 | IL11 | na | 34460 | -0.537 | -0.0639 | Yes |
| 252 | OSMR | na | 34470 | -0.540 | -0.0512 | Yes |
| 253 | IL10RA | na | 34529 | -0.565 | -0.0393 | Yes |
| 254 | CXCL10 | na | 34557 | -0.586 | -0.0260 | Yes |
| 255 | IL22RA2 | na | 34572 | -0.598 | -0.0120 | Yes |
| 256 | LTB | na | 34656 | -0.687 | 0.0021 | Yes |
Table: GSEA details [plain text format]

  

Fig 2: KEGG\_CYTOKINE\_CYTOKINE\_RECEPTOR\_INTERACTION      
 Blue-Pink O' Gram in the Space of the Analyzed GeneSet

  

Fig 3: KEGG\_CYTOKINE\_CYTOKINE\_RECEPTOR\_INTERACTION: Random ES distribution      
 Gene set null distribution of ES for **KEGG\_CYTOKINE\_CYTOKINE\_RECEPTOR\_INTERACTION**
